# The Diverse Roles of FLOWERING LOCUS C in Annual and Perennial Brassicaceae Species

**DOI:** 10.3389/fpls.2021.627258

**Published:** 2021-02-15

**Authors:** Wim J. J. Soppe, Natanael Viñegra de la Torre, Maria C. Albani

**Affiliations:** ^1^Rijk Zwaan, De Lier, Netherlands; ^2^Institute for Plant Sciences, University of Cologne, Cologne, Germany; ^3^Max Planck Institute for Plant Breeding Research, Cologne, Germany; ^4^Cluster of Excellence on Plant Sciences, “SMART Plants for Tomorrow’s Needs,” Heinrich Heine University Düsseldorf, Düsseldorf, Germany

**Keywords:** *Arabis alpina*, FLC, flowering, PEP1, perennial, perpetual flowering, polycarpic growth habit, vernalization

## Abstract

Most temperate species require prolonged exposure to winter chilling temperatures to flower in the spring. In the Brassicaceae, the MADS box transcription factor FLOWERING LOCUS C (FLC) is a major regulator of flowering in response to prolonged cold exposure, a process called vernalization. Winter annual *Arabidopsis thaliana* accessions initiate flowering in the spring due to the stable silencing of *FLC* by vernalization. The role of FLC has also been explored in perennials within the Brassicaceae family, such as *Arabis alpina.* The flowering pattern in *A. alpina* differs from the one in *A. thaliana*. *A. alpina* plants initiate flower buds during vernalization but only flower after subsequent exposure to growth-promoting conditions. Here we discuss the role of FLC in annual and perennial Brassicaceae species. We show that, besides its conserved role in flowering, FLC has acquired additional functions that contribute to vegetative and seed traits. *PERPETUAL FLOWERING 1* (*PEP1*), the *A. alpina FLC* ortholog, contributes to the perennial growth habit. We discuss that PEP1 directly and indirectly, regulates traits such as the duration of the flowering episode, polycarpic growth habit and shoot architecture. We suggest that these additional roles of *PEP1* are facilitated by (1) the ability of *A. alpina* plants to form flower buds during long-term cold exposure, (2) age-related differences between meristems, which enable that not all meristems initiate flowering during cold exposure, and (3) differences between meristems in stable silencing of *PEP1* after long-term cold, which ensure that *PEP1* expression levels will remain low after vernalization only in meristems that commit to flowering during cold exposure. These features result in spatiotemporal seasonal changes of *PEP1* expression during the *A. alpina* life cycle that contribute to the perennial growth habit. FLC and PEP1 have also been shown to influence the timing of another developmental transition in the plant, seed germination, by influencing seed dormancy and longevity. This suggests that during evolution, *FLC* and its orthologs adopted both similar and divergent roles to regulate life history traits. Spatiotemporal changes of *FLC* transcript accumulation drive developmental decisions and contribute to life history evolution.

## Plants Have Different Ways of Using Winter Cold to Synchronize Flowering in the Spring

Winter annuals, biennials and perennial species overwinter as seedlings or plants and flower in the spring when favorable environmental conditions return. Annual and biennial species follow a monocarpic life strategy and die after setting seeds once, whereas the majority of perennials are polycarpic and are able to reproduce several times during their life time (Albani and Coupland, 2010; Bratzel and Turck, 2015). Low temperatures during the winter are important to enable synchronous flowering in the spring but generally regulate different stages of the flowering process in monocarpic compared to polycarpic species.

**FIGURE 1:**
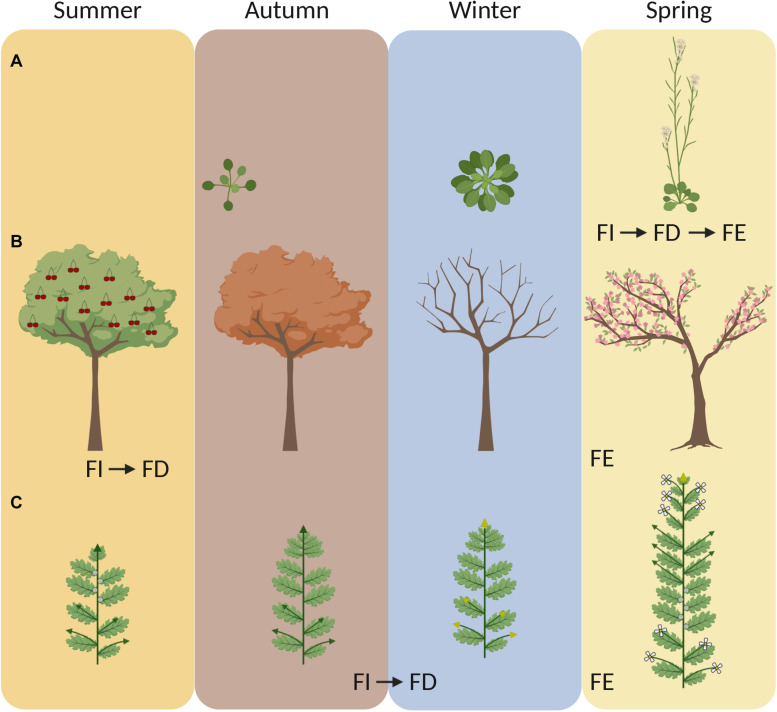
Annuals and perennials initiate floral buds at different times during the year and use winter to synchronize flowering. Cartoons depict the yearly life cycle of **(A)** a winter annual (e.g., *A. thaliana*), **(B)** a woody perennial (e.g., cherry tree) and **(C)** an herbaceous alpine perennial (e.g., *A. alpina*). Winter annual *A. thaliana* accessions induce flowering in the spring. After floral induction (FI), flowering is initiated and floral buds develop (FD) and emerge (FE). In *A. thaliana*, all steps from floral induction to emergence are very rapid (FI, FD and FE). In perennials, the stages of floral induction (FI) and floral emergence (FE) are temporarily separated. Many woody perennials induce and initiate flowering during summer (FI and FD) but flower buds emerge (FE) in the spring of the next year. In *A. alpina*, flowering is induced and initiated (FI and FD) during cold exposure in the late autumn, but flowers emerge (FE) after plants return to warm temperatures. In panel **(C)**, *green arrows* represent vegetative branches and *yellow arrows* represent flowering branches. Image created with BioRender.com.

Winter annuals and biennials require prolonged exposure to cold to accelerate or to enable flowering, a process called vernalization. Prolonged cold exposure is effective when it is applied to imbibed seeds or young seedlings (of winter annual species), or to older plants (of winter annual and biennial species) (Lang, 1952; Chouard, 1960). The requirement of cold exposure for flowering is common in plants adapted to temperate climates and ensures that they will not flower before the winter (Chouard, 1960). Vernalization is considered as a preparatory process that has to take place before a plant can initiate flowering in response to increased daylength in the spring (Chouard, 1960). The effectiveness of vernalization is usually estimated by the reduction of days that plants take to flower after returning to growth-promoting conditions (Chouard, 1960). Thus, vernalization aligns with the life cycle of winter annual and biennial species that initiate flowering in the spring (Figure 1A; Chouard, 1960). In *A. thaliana*, vernalization has also a quantitative effect on flowering and the duration of cold that is required for the acceleration of flowering can vary between accessions (Shindo et al., 2006; Li et al., 2014). Plants can be vernalized at different temperatures ranging from 0 to 16°C (Wollenberg and Amasino, 2012; Duncan et al., 2015). In some habitats, this temperature range can be achieved during summer or autumn, suggesting that plants can be vernalized before the winter (Billings and Mooney, 1968; Duncan et al., 2015; O’Neill et al., 2019). In addition, older plants show a stronger response to vernalization than young seedlings, suggesting that the developmental stage/age of the plant influences its ability to respond to prolonged cold treatment (Chouard, 1960). The effect of cold exposure is also reversible and plants can devernalize when cold exposure is followed by high temperatures (Chouard, 1960; Périlleux et al., 2013). This suggests that the flower-promoting role of vernalization can be lost when plants are exposed to exceptionally high spring temperatures.

Polycarpic perennials do not commit all meristems to flowering and are able to maintain growth from one year to the next by keeping some meristems in a vegetative state (Albani and Coupland, 2010). Maintenance of vegetative growth after flowering is determined by the coordinated action of age-related factors, which determine whether individual meristems are sensitive to flower-inductive stimuli, and seasonal changes in expression patterns of environmentally-regulated flowering time genes (Wang et al., 2009; Bergonzi and Albani, 2011; Koskela et al., 2012; Hyun et al., 2017, 2019; Zhou et al., 2021). Perennials, however, still have to synchronize their annual cycle in order to flower and set fruits during favorable environmental conditions in spring and summer (Falavigna et al., 2019). The environmental conditions that induce flowering in temperate perennials vary greatly between species, although most of them concentrate their flowering season in the spring. For example, grapevine, sweet cherry and peach trees initiate flower buds during the summer (Figure 1B; Engin and Ünal, 2007; Carmona et al., 2008; Vimont et al., 2019). Therefore, spring flowering in these perennials occurs when the flower buds, which were initiated the previous year, grow out. Interestingly, the observed variation in spring flowering between cultivars is not associated with differences in flower bud initiation but rather with the requirement of prolonged cold exposure that is needed for plants to exit the endodormant state that they enter in autumn (Vimont et al., 2019). In general, bud dormancy can be divided into three phases: paradormancy, endodormancy and ecodormancy. Paradormancy (also referred as latency) is the stage during which the growth of a bud is inhibited by surrounding organs (Lang et al., 1987). The other stages of dormancy are environmentally regulated, with endodormancy being induced and ecodormancy being maintained by environmental cues (Lang et al., 1987). Buds enter endodormancy during the autumn and throughout this phase their growth is inhibited by internal signals (Lang et al., 1987). Prolonged exposure to low temperatures in the winter is required for endodormancy release, and once the chilling requirement is met the buds transition into ecodormancy (reviewed in Falavigna et al., 2019). Bud growth during ecodormancy is inhibited by unfavorable environmental conditions and can be reactivated again when plants experience growth-promoting conditions (Lang et al., 1987). Overall, prolonged cold exposure is important for bud dormancy release and synchronized resumption of growth the following spring (referred to as budbreak). Exposure to chilling temperatures for dormancy breaking is considered to be a distinct process from vernalization (Chouard, 1960). Specifically, because low temperatures during dormancy breaking do not induce a phase change that leads to the formation of new kinds of organs but cause the regrowth of already existing organs (Chouard, 1960). However, there are obvious similarities between both processes, which both require long-term cold to synchronize spring flowering (Or, 2009; Atkinson et al., 2013) and share a considerable overlap in their molecular pathways (reviewed in Horvath, 2009).

**FIGURE 2:**
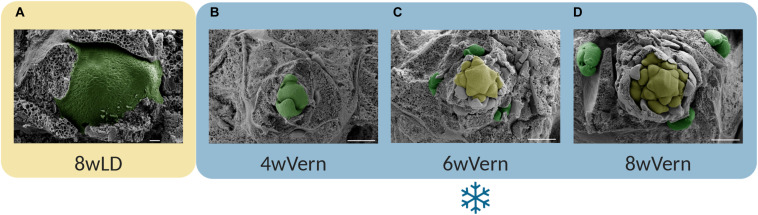
Flower buds in *A. alpina* are initiated during vernalization. Progression of the shoot apical meristem of *A. alpina* accession Pajares imaged by *scanning electron microscopy* (*SEM*) in **(A)** plants were grown for 8 weeks in a long day greenhouse, and subsequently transferred to vernalization for **(B)** 4 weeks (4wVern), **(C)** 6 weeks (6wVern) or **(D)** 8 weeks (8wVern). The main shoot apex is vegetative in plants grown in greenhouse conditions or vernalized only for 4 weeks (4wVern) (**A,B**, highlighted in *green*). The main shoot apex initiates flowering after 6 weeks in vernalization (**C**, highlighted in *yellow*) and inflorescences develop during vernalization (**D**, highlighted in *yellow*). In the axils of leaf primordia close to the shoot apical meristem, new vegetative buds are also formed (**C,D**, highlighted in *green*). Scale bars correspond to 20 μm **(A)** or to 200 μm **(B–D)**. Colored boxes represent different growth conditions: the *yellow box* a long day greenhouse; and the *blue box* the vernalization at 4°C and short days (8 h light: 16 h dark). SEM pictures were modified from the Ph.D. dissertation of Zhou (2019). Image was composed using BioRender.com.

There is also a third variant by which low temperatures lead to flowering. This variant has been observed in several Brassicaceae species, including winter oil seed rape, *A. alpina*, *Arabidopsis lyrata*, pak choi, and Brussels sprouts (Chouard, 1960; Wang et al., 2009; Kemi et al., 2019; O’Neill et al., 2019). In these species, flower buds initiate and develop during cold exposure, suggesting that vernalization is not only a preparatory process for flowering but also directly regulates the initiation and formation of floral buds (Figure 1C; Chouard, 1960; Wang et al., 2009; Kemi et al., 2019; O’Neill et al., 2019). In the perennial *A. alpina*, flowering is initiated during vernalization under short photoperiods (Figures 1C, 2; Wang et al., 2009; Lazaro et al., 2018). Similarly, field studies in winter oil seed rape demonstrated that inflorescences are initiated during the autumn in response to low temperatures (O’Neill et al., 2019). In both species, the length of the cold exposure is also important for the outgrowth of flower buds after the return to warm temperatures (Lazaro et al., 2018; O’Neill et al., 2019). Flower buds may still enter dormancy during the winter or grow more slowly due to low temperatures. In field studies using oil seed rape plants, dissection of shoot apical meristems showed that inflorescence meristems gradually grow during the winter and thus may not be dormant (O’Neill et al., 2019). Although flower buds in such species develop during autumn, daylength is still important for flowering. As shown in *A. lyrata*, long photoperiods are important for inflorescence bolting in the spring (Kemi et al., 2019). Ecological studies in alpine species suggest that flower bud development during cold exposure can be an advantageous adaptive trait in harsh environments characterized by prolonged snow coverage and short growing seasons (Diggle, 1997; Meloche and Diggle, 2001). This modification of the role of vernalization on flowering does not seem to be related to the plant life strategy as both annual and perennial species have been reported to form flower buds during cold treatment (Chouard, 1960; Wang et al., 2009; Kemi et al., 2019; O’Neill et al., 2019).

## The Role and Regulation of *FLOWERING LOCUS C* (*FLC*) in *A. thaliana*

Several studies in *A. thaliana* demonstrated that the major regulator of flowering through the vernalization pathway is the MADS box transcription factor FLOWERING LOCUS C (FLC) (Michaels and Amasino, 1999; Sheldon et al., 2000). In temperate grasses, which also require prolonged cold exposure to flower, and also in biennials such as sugar beet, the regulation of vernalization is mediated by pathways independent of FLC (reviewed in Bouché et al., 2017). FLC is a floral repressor that is highly expressed in *A. thaliana* accessions that require vernalization to accelerate flowering (Shindo et al., 2006; Li et al., 2014). Prolonged exposure to low temperatures regulates *FLC* in two ways: (1) by quantitatively repressing *FLC* transcript accumulation and (2) by ensuring that *FLC* will remain stably silenced after plants have returned to warm conditions. The major mechanism that drives *FLC* transcriptional regulation by long-term cold consists of chromatin modifications (reviewed in Berry and Dean, 2015). In *A. thaliana* accessions, non-coding *cis* polymorphisms at the *FLC* locus underlie variation in the length of cold required to achieve stable silencing of *FLC* (Coustham et al., 2012; Qüesta et al., 2020). *FLC* is transcriptionally repressed by long non-coding RNAs (lncRNAs), expressed at the locus in response to cold, which play an early role in the epigenetic silencing of *FLC* (Heo and Sung, 2011; Csorba et al., 2014). A parallel pathway that interprets long-term exposure to low temperatures involves the plant homeodomain (PHD) protein, VERNALIZATION INSENSITIVE 3 (VIN3) (Sung and Amasino, 2004; Wood et al., 2006; De Lucia et al., 2008; Swiezewski et al., 2009). *VIN3* expression increases gradually during cold by a mechanism that is mediated by the NAC transcription factor NTM1-LIKE 8 (NTL8) (Sung and Amasino, 2004; Bond et al., 2009; Zhao et al., 2020). NTL8 protein accumulates during cold exposure, due to growth retardation and reduced number of cell divisions that occur at low temperatures, and upregulates *VIN3* transcription (Zhao et al., 2020). Subsequently, VIN3 forms a complex with its homologous PHD protein VERNALIZATION5 (VIN5) and the Polycomb Repressive Complex 2 (PRC2) proteins VERNALIZATION2 (VRN2), MSI1, FIE/EED, and the E(z) homologs SWINGER (SWN) and CURLY LEAF (CLF) (reviewed in Berry and Dean, 2015). This PHD-PRC2 complex is recruited to the *FLC* locus at 3 nucleosomes, covering exon 1 and part of the first intron (which is called the nucleation region), and methylates H3 lysine 27 residues resulting in a gradual cell-autonomous *FLC* silencing (Angel et al., 2011). The B3-binding transcription factor VP1/ABI3-LIKE 1 (VAL1) is required for the PHD-PRC2 action at *FLC* (Qüesta et al., 2016; Yuan et al., 2016). After cold exposure, the H3K27me3 mark spreads over the entire *FLC* locus and ensures maintenance of long-term silencing of *FLC* (Finnegan and Dennis, 2007; De Lucia et al., 2008). This spreading of H3K27me3 is enabled by the PHD-PRC2 complex and requires the action of the Polycomb protein LIKE HETEROCHROMATIN PROTEIN1 (LHP1) that specifically binds to H3K27me3 marks (reviewed in Costa and Dean, 2019).

Similar to other MADS-domain proteins, FLC regulates its downstream genes by binding to conserved CArG-box motifs in their promoters or introns (Deng et al., 2011; Mateos et al., 2017). FLC target genes act in different pathways throughout development, of which several are implicated in flowering (Deng et al., 2011; Mateos et al., 2017). These include the floral promoter *FLOWERING LOCUS T* (*FT*), that regulates flowering through the photoperiod pathway; *SUPPRESSOR OF OVEREXPRESSION OF CONSTANS 1* (*SOC1*), that regulates several genes involved in floral transition at the shoot apex; the SQUAMOSA BINDING PROTEIN LIKE (SPL) family member SPL15, that contributes to reproductive competence and floral transition; and *SEPALLATA3* (*SEP3*), that regulates flower development (reviewed in Madrid et al., 2020). Stable *FLC* silencing after vernalization is, therefore, a key feature to explain the flowering pattern of winter annual *A. thaliana* accessions. This is because the reduced mRNA levels of *FLC* allow the activation of floral promoting pathways that ensure the initiation of flowering in the spring. For the life cycle of an annual plant it is important that *FLC* expression is restored in the next generation. In *A. thaliana* this occurs during embryo development and by the end of embryogenesis all H3K27me3 marks have been removed from the *FLC* locus so that the gene is fully reactivated (Tao et al., 2019; Luo et al., 2020).

*Arabidopsis thaliana* plants that carry weak *FLC* alleles can initiate flowering in the autumn due to low starting *FLC* mRNA levels (Hepworth et al., 2020). These genotypes bolt precociously and show higher mortality in the field, which emphasizes the importance of *FLC* in ensuring spring flowering in *A. thaliana* (Hepworth et al., 2020). In oil seed rape plants, which initiate flowering during cold exposure, the starting levels of *BnaFLC* also negatively correlate with the timing of flower bud initiation in the autumn (O’Neill et al., 2019). Cultivars with lower *BnaFLC* mRNA levels initiate flowering earlier compared to cultivars with higher starting *BnaFLC* mRNA levels (O’Neill et al., 2019). Interestingly, although oil seed rape plants do not bolt precociously, there is a penalty on plant yield if flowering is initiated earlier in the autumn (Brown et al., 2019).

## The Role of FLC in the Regulation of Flowering in Perennial Brassicaceae Species

The role of FLC has been explored in perennial Brassicaceae species such as *A. alpina* (Wang et al., 2009; Albani et al., 2012), *A. lyrata* (Kemi et al., 2013), *Arabidopsis halleri* (Aikawa et al., 2010), *Arabidopsis arenosa* (Baduel et al., 2016), and *Boechera stricta* (Lee et al., 2018). At regions, syntenic to the *FLC* locus in *A. thaliana*, some of these species contain tandem *FLC* copies derived from duplication events after their divergence from *A. thaliana* (Nah and Chen, 2010; Albani et al., 2012). *A. lyrata* has two tandemly duplicated *FLC* genes (*FLC1* and *FLC2*), whereas *A. arenosa* contains one partial and two complete *FLC* copies (Nah and Chen, 2010). In *A. alpina* a region of ∼2 kb at the *FLC* ortholog, *PERPETUAL FLOWERING 1* (*PEP1*), has been tandemly duplicated (Albani et al., 2012). This tandem duplication includes the first exon of *PEP1* (*AaFLC*) and parts of its promoter and first intron (Albani et al., 2012). Interestingly, this tandem duplication leads to the production of two overlapping *PEP1* transcripts (*PEP1a* and *PEP1b*) that have different transcriptional start sites, using the two tandemly duplicated first exons (Albani et al., 2012). This finding could suggest that duplication events in *FLC* orthologs may give rise to diverse functions or specialized forms of the protein. Nevertheless, tandem duplications in *FLC* do not contribute to the perennial life cycle. For example, several *FLC* orthologs in both annual and perennial Arabis species contain duplicated regions (Kiefer et al., 2017). In addition, even within *A. alpina* not all accessions contain the partial duplication at *PEP1* (*AaFLC*) (Albani et al., 2012).

The role of FLC in flowering in perennial Brassicaceae is similar to that reported in *A. thaliana* and other annual Brassicas. *FLC1* mRNA levels in *A. lyrata* are down regulated in response to vernalization and *FLC* genes are co-localized with QTLs determining flowering time differences between late and early flowering *A. lyrata* accessions (Kemi et al., 2013). Similarly, *A. alpina* accessions carrying lesions in *PEP1* (*AaFLC*) do not require vernalization to flower (Albani et al., 2012). The *A. alpina pep1* mutant also flowers without vernalization compared to its wild type accession Pajares, that has an obligate vernalization requirement to flower (Wang et al., 2009). An interesting feature in perennial Brassicaceae is that *FLC* is not stably silenced after vernalization, resembling *FLC* expression patterns of *A. thaliana* accessions that require extended vernalization to flower (Wang et al., 2009; Kemi et al., 2013; Baduel et al., 2016; Qüesta et al., 2020). Unstable silencing of *FLC* orthologs by vernalization has been observed in plants grown in controlled environmental conditions (Wang et al., 2009; Kemi et al., 2013; Lazaro et al., 2018) and also in plants experiencing winter chilling temperatures in the field (Aikawa et al., 2010; Nishio et al., 2020). For instance, transcript accumulation of the *A. halleri FLC* (*AhgFLC*) is reduced during winter and is upregulated again the following spring (Aikawa et al., 2010). Interestingly, the accumulation of the H3K27me3 mark mirrors the expression patterns of *AhgFLC* (Nishio et al., 2020). In *A. alpina* plants vernalized for 12 weeks, *PEP1* (*AaFLC*) is also temporarily silenced during cold exposure but it is transcriptionally activated again after plants return to growth-promoting conditions (Wang et al., 2009). On the contrary, *FLC* of the annual close relative *Arabis montbretiana* (*AmFLC*) is stably silenced by vernalization (Kiefer et al., 2017). Plants carrying introgressions of genomic segments containing the *AmFLC* into *A. alpina*, indicated that, in the same genetic background, *PEP1* and *AmFLC* are differentially silenced after vernalization (Kiefer et al., 2017; Hyun et al., 2019). The major difference between the *A. alpina* and *A. montbretiana FLC* orthologs lies in polymorphisms within non-coding regions suggesting that, similar to *A. thaliana FLC*, non-coding polymorphisms may confer differences between *PEP1* and *AmFLC* in stable silencing by vernalization (Coustham et al., 2012; Kiefer et al., 2017). Within the Arabideae, the annual life strategy arose several times during evolution (Kiefer et al., 2017). Phylogenetic studies in different taxa suggest that annuals were derived from perennial ancestors, although in some genera annuals seem to have switched back to the perennial growth habit (Friedman and Rubin, 2015). We can, therefore, hypothesize that Brassicaceae annual species may have gained independently *cis* polymorphisms at *FLC* non-coding regions to ensure stable silencing, which is important for the spring-flowering habit.

In addition to the seasonal cycling of *PEP1* mRNA levels, the perennial behavior in *A. alpina* is also characterized by a strong regulation of age-related factors that determine whether individual meristems will initiate flowering in response to vernalization or not (Wang et al., 2009; Bergonzi et al., 2013; Hyun et al., 2019; Lazaro et al., 2019; Zhou et al., 2021). Interestingly, the degree of *PEP1* (*AaFLC*) stable silencing by vernalization has been demonstrated to vary between meristems, depending on whether they initiated flowering during the cold exposure (Lazaro et al., 2018). In juvenile meristems that fail to initiate flowering during cold treatment, *PEP1* (*AaFLC*) mRNA levels are reduced during vernalization and upregulated again after plants return to warm temperatures (Wang et al., 2011; Lazaro et al., 2018). In this way, *PEP1* (*AaFLC*) ensures maintenance of vegetative growth after vernalization even when meristems develop further and acquire competence to flower. The underlying cause of the difference in *PEP1* (*AaFLC*) regulation by prolonged cold exposure between juvenile and adult meristems is not known. In *A. thaliana*, DNA replication has been proposed to be essential for the maintenance of *FLC* silencing after vernalization (Finnegan and Dennis, 2007; Qüesta et al., 2020). On the same lines, differences in cell division and DNA replication between meristems may explain the variation observed in *PEP1* (*AaFLC*) stable silencing by cold. In *A. alpina*, *PEP1* (*AaFLC*) reactivation after vernalization has been shown to be facilitated by *PERPETUAL FLOWERING2* [*PEP2*, the *A. alpina* ortholog of *APETALA2* (*AP2*)], a role not previously reported for AP2 in *A. thaliana* (Bergonzi et al., 2013; Lazaro et al., 2019). The mechanism for this role of PEP2 (AaAP2) is not known. AP2 in *A. thaliana* interacts with chromatin remodeling factors such as HISTONE DEACETYLASE 19 (HDA19) (Krogan et al., 2012), but has never been associated with histone demethylases which could facilitate the upregulation of *PEP1* (*AaFLC*) mRNA levels after vernalization.

Flowering in adult/competent meristems is initiated at about 6 weeks after the start of vernalization (Figure 2; Wang et al., 2009; Lazaro et al., 2018). For plants to flower, however, exposure to a minimum of 12 weeks of vernalization is required as inflorescence meristems should develop further during vernalization (Wang et al., 2009; Lazaro et al., 2018). The molecular mechanisms enabling floral development during vernalization involve the floral integrator AaSPL15 and increased sensitivity to the growth regulator gibberellin (GA) (Hyun et al., 2019; Tilmes et al., 2019). *PEP1* expression is still not stably silenced after 12 weeks of vernalization, which leads to floral reversion phenotypes, such as the presence of bracts or flower to inflorescence reversion (Wang et al., 2009; Lazaro et al., 2018). Exposure to a minimum of 18 weeks of cold treatment is required to inhibit floral reversion and to stably silence *PEP1* (Lazaro et al., 2018). Floral reversion phenotypes are also observed in *A. halleri* plants, although in this species it has not been reported whether flower buds are formed the previous year (Aikawa et al., 2010; Nishio et al., 2020).

Interestingly, *A. alpina* genes that act in the age pathway to regulate the age-dependent response to vernalization also influence the duration of vernalization required for flowering. For example, PEP2, TARGET OF EAT2 (AaTOE2), TERMINAL FLOWER1 (AaTFL1), and AaSPL15 determine the age at which plants become competent to flower in response to cold treatment, but also control the duration of vernalization required for flowering (Wang et al., 2011; Lazaro et al., 2018; Hyun et al., 2019; Zhou et al., 2021). The *pep2* and *Aatoe2* mutants and transgenic lines with reduced function of AaTFL1 (DsRNAi *AaTFL1*) flower when vernalized at a young age (at 3 weeks old compared to 5 weeks for wild type plants) and require less than 8 weeks of vernalization to flower (compared to 12 weeks for wild type plants) (Wang et al., 2011; Lazaro et al., 2019; Zhou et al., 2021).

In woody perennials, bud dormancy is regulated by a cluster of tandemly duplicated genes, *DORMANCY ASSOCIATED MADS-BOX* 1-6 (*DAM1-6*) (reviewed in Falavigna et al., 2019). Similar to *FLC*, *DAM* genes encode MADS box transcription factors, but these are more closely related to *A. thaliana* SHORT VEGETATIVE PHASE (SVP) and AGAMOUS-like 24 (AGL24) (reviewed in Falavigna et al., 2019). The expression of *DAM* genes is upregulated during dormancy induction and downregulated during the transition from endo- to ecodormancy (Maurya and Bhalerao, 2017; Vimont et al., 2019). *DAM* genes are also regulated by epigenetic mechanisms such as histone modifications (Leida et al., 2012; de la Fuente et al., 2015; Saito et al., 2015) and DNA methylation (Rothkegel et al., 2017). In *A. thaliana*, SVP is a floral repressor that interacts with other transcription factors to regulate floral development and flowering time (De Folter et al., 2005; Gregis et al., 2006; Lee et al., 2007; Balanzà et al., 2014; Mateos et al., 2015). FLC is an interactor of SVP *in A. thaliana* and both proteins act together to regulate flowering time by repressing FT and SOC1 or GA-related genes (Lee et al., 2007; Andrés et al., 2014; Mateos et al., 2015). These data suggest that SVP or other MADS box genes might have taken the role of FLC in woody perennials to regulate developmental traits related to prolonged cold exposure.

## Pleiotropic Roles of FLC in Perennial Brassicaceae

In addition to the role of FLC in flowering, studies in *A. thaliana* and other Brassicaceae species demonstrated that FLC has pleiotropic effects on other developmental traits. Pleiotropic phenotypes may be an indirect effect caused by differences in flowering behavior adopted by different species and/or may be due to direct roles of FLC on other traits. Examples of direct and indirect pleiotropic effects of FLC are reported below.

### Polycarpic Growth Habit and Shoot Architecture in the Perennial *A. alpina*

**FIGURE 3:**
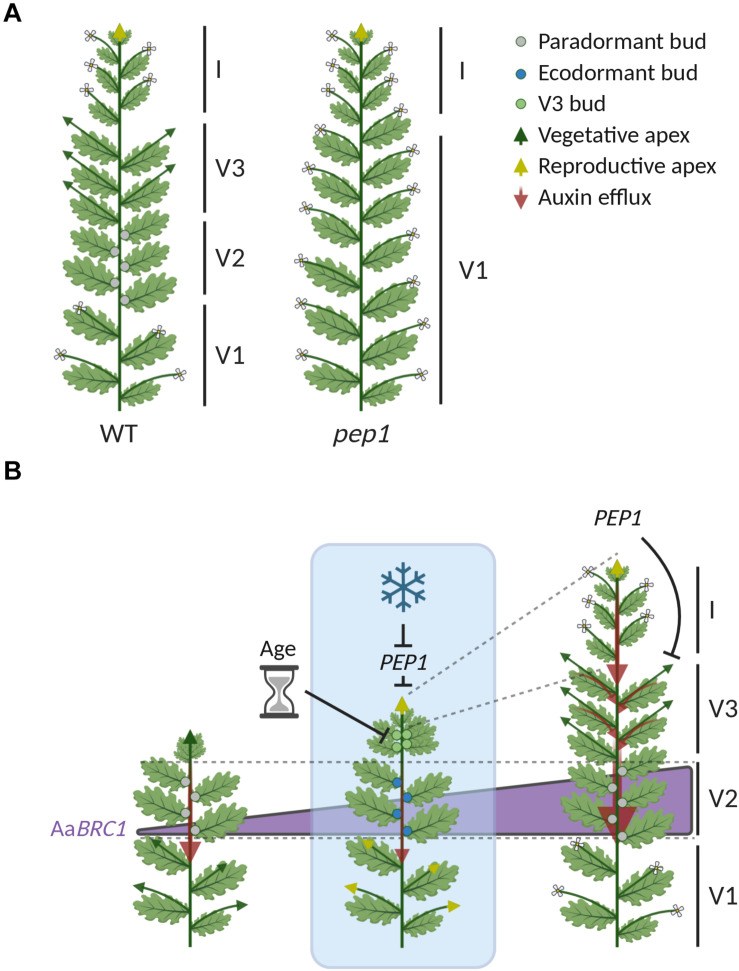
Shoot architecture in *A. alpina* is shaped by vernalization and *PEP1*. **(A)** Representation of wild type *A. alpina* (WT) and *pep1* mutant plants after flowering. Wild type plants require exposure to long-term cold to flower, whereas *pep1* mutants do not and can flower directly in greenhouse conditions. Flowering wild type plants have a complex shoot architecture, which is organized in differentiated zones of basal flowering axillary branches (V1) that partially senesce; dormant buds (V2); vegetative axillary branches (V3); and the inflorescence (I). The *pep1* mutant lacks this zonation and has only V1 axillary branches and the inflorescence. **(B)** Representation of the shoot architecture of a wild type plant during its life cycle before, during and after vernalization. *Blue box* represents exposure to prolonged cold, whereas the time before and after cold exposure is not colored. During long-term cold, *PEP1* (*AaFLC*) transcript levels are reduced and both the main shoot apex and the apices of V1 axillary branches initiate flowering (*yellow arrowheads*). In addition, new buds (V3 buds, *green circles*) are formed in those leaf primordia close to the main shoot apex. These V3 buds (*green circles*) do not initiate flowering during cold exposure due to the repressive action of the age pathway. After cold exposure, V3 buds give rise to V3 axillary branches, which grow vegetatively because *PEP1* (*AaFLC*) transcript levels are upregulated and repress flowering. The outgrowth of V2 buds is inhibited by paradormancy (or apical dominance) before vernalization (*gray circles*), by ecodormancy during cold exposure (*blue circles*) and by paradormancy (or latency) after plants return to growth-promoting conditions (*gray circles*). The dormancy status of V2 buds increases during and after vernalization, and is regulated by a mechanism that is independent of PEP1 (AaFLC). Increased dormancy in V2 buds correlates with the upregulation of *BRANCHED 1* (*AaBRC1*), a repressor of bud outgrowth (*illustrated with the purple triangle*). After vernalization, increased dormancy in V2 buds is correlated with increased endogenous IAA levels in the stem and auxin transport (*red arrows*). Image created using BioRender.com.

*PERPETUAL FLOWERING 1* (AaFLC) contributes to the polycarpic growth habit and ensures that *A. alpina* plants maintain vegetative growth after flowering (Wang et al., 2009). In *pep1* mutants all axillary branches flower, suggesting that PEP1 (AaFLC) regulates the fate of axillary meristems (Wang et al., 2009; Vayssières et al., 2020). This role of PEP1 (AaFLC) is facilitated by the ability of *A. alpina* plants to initiate flowering during vernalization (Figure 2; Wang et al., 2009; Lazaro et al., 2018). Flower bud development during vernalization is also coupled with the formation of axillary meristems in the leaf axils close to the shoot apical meristem (Wang et al., 2009; Ponraj and Theres, 2020; Vayssières et al., 2020). These newly formed axillary meristems develop into buds in a basipetal sequence and do not commit to reproductive development during cold exposure (*green* in Figures 2C,D; Wang et al., 2009; Ponraj and Theres, 2020; Vayssières et al., 2020). *A. alpina* mutants that carry lesions in floral repressors in the age pathway (AaTOE2 and AaAP2/PEP2) but also transgenic lines with altered levels of SPLs (e.g., microRNA cleavage-resistant forms of SPL15, rSPL15) have axillary branches at these subapical nodes which develop and become reproductive (Hyun et al., 2019; Lazaro et al., 2019; Zhou et al., 2021). A closer look at the *A. alpina toe2* mutant demonstrated that these subapical buds initiate flowering already during vernalization (Figure 3B; Zhou et al., 2021). This result suggests that the fate of the subapical buds is determined during vernalization by the age pathway.

Wild type *A. alpina* flowering plants exhibit a complex shoot architecture in which axillary meristems behave in different ways and are organized in different zones (Figure 3A; Lazaro et al., 2018; Ponraj and Theres, 2020; Vayssières et al., 2020). Zonation patterns of differential bud activity and fate have also been reported in other perennials (Costes et al., 2014). The main stem of *A. alpina* plants consist of zones of basal axillary flowering branches (V1), axillary vegetative branches (V3) and dormant buds (V2) (Figure 3A; Lazaro et al., 2018; Ponraj and Theres, 2020; Vayssières et al., 2020). PEP1 (AaFLC) contributes to this complex shoot architecture considering that the *pep1* mutant consists only of V1 axillary branches and lacks the V2 dormant buds and V3 vegetative branches (Figure 3A; Wang et al., 2009; Vayssières et al., 2020). In wild type plants, V1 axillary branches behave similarly to the main shoot apex and initiate flowering during vernalization, when *PEP1* mRNA levels are reduced (*yellow arrows* in Figure 3B; Wang et al., 2009; Vayssières et al., 2020). V3 axillary branches arise after vernalization from buds formed at the subapical nodes during cold (*green* in Figures 2C,D; *green circles* in Figure 3B; Wang et al., 2009; Lazaro et al., 2018; Ponraj and Theres, 2020; Vayssières et al., 2020; Zhou et al., 2021). PEP1 determines the fate of these buds after vernalization (Wang et al., 2009; Lazaro et al., 2018). Specifically, *PEP1* mRNA levels are upregulated after vernalization to repress flowering and ensure that these branches will maintain vegetative development (V3 *green arrows* in Figure 3B; Wang et al., 2009; Lazaro et al., 2018). V2 buds follow a different developmental path, in which their growth is repressed before and after vernalization by paradormancy (or latency) and their activity is influenced by their position on the shoot (*gray circles* in Figure 3B; Vayssières et al., 2020). During cold exposure, V2 buds become ecodormant (*blue circles* in Figure 3B; Vayssières et al., 2020). The activity of V2 buds is not directly regulated by PEP1 (AaFLC) but rather by the *A. alpina* ortholog of *BRANCHED 1* (*AaBRC1*), whose expression increases during and after vernalization (*Purple triangle* in Figure 3B; Vayssières et al., 2020). Flowering time genes such as FT in *A. thaliana* and SPL15 (OsSPL14) in rice have been reported to influence bud activity (Miura et al., 2010; Tsuji et al., 2015). PEP1 (AaFLC) might indirectly inhibit the outgrowth of buds in the nodes within the V2 zone. Specifically, PEP1 (AaFLC), by repressing flowering in V3 axillary branches, ensures the continuous formation of young leaves that may act as an auxin source to inhibit the outgrowth of V2 buds (*Red arrows* in Figure 3B; Vayssières et al., 2020). Many studies in *A. thaliana* support the theory that auxin saturation in the transport stream of the main stem can inhibit bud outgrowth by blocking auxin transport from lateral sources (Prusinkiewicz et al., 2009). Dormant buds are always located at the axils of leaves below the zone of V3 vegetative branches, supporting the hypothesis that V3 branches may become the new auxin source that represses the outgrowth of V2 buds (*Red arrows* in Figure 3B; Vayssières et al., 2020). Nevertheless, mutants in which V3 branches flower still have a dormant bud zone (Hyun et al., 2019; Zhou et al., 2021). This phenotype can be explained by the fact that flowering V3 branches still maintain vegetative development through secondary or tertiary branching and can still act as auxin sources (Zhou et al., 2021).

Overall, the complex architecture in *A. alpina* is a result of the effect of prolonged cold exposure on the regulation of flowering and on bud dormancy, which differs between meristems. These roles of cold are partially controlled by PEP1 (AaFLC).

### Duration of the Flowering Season in the Perennial *A. alpina*

Many temperate perennials restrict the duration of the flowering season to spring and summer. In *A. alpina, pep1* mutants and accessions that carry inactive *PEP1* alleles flower perpetually (continuously) (Wang et al., 2009; Albani et al., 2012). In the field, perpetual flowering genotypes with non-functional *PEP1* alleles show extended and asynchronous flowering (Hughes et al., 2019). Interestingly, these genotypes also exhibit reduced survival in the field, suggesting that PEP1 contributes to plant fitness (Hughes et al., 2019). The role of PEP1 on the duration of the flowering season is a result of the upregulation of *PEP1* mRNA levels after vernalization. PEP1 restricts the duration of flowering season by ensuring that no further meristems will initiate flowering after the return to warm temperatures.

So far, there are only a few studies exploring the regulation of flowering duration in other perennials. In the Rosaceace, there is natural variation for this trait. Genotypes that follow either the seasonal flowering habit (and restrict the duration of the flowering episode) or the perpetual flowering habit (and flower continuously) can be found (Brown and Wareing, 1965; Crespel et al., 2002; Albani et al., 2004). In the wild strawberry *Fragaria vesca* and rose, the perpetual flowering habit arose from loss of function mutations in the floral repressor TERMINAL FLOWER1 (TFL1) (Iwata et al., 2012; Koskela et al., 2012). Interestingly, all perpetual flowering *F. vesca* accessions contain the same 2 bp deletion in *FvTFL1*, suggesting that in *F. vesca* (but not in *A. alpina* and rose) the perpetual flowering habit arose only once during evolution (Albani et al., 2012; Iwata et al., 2012; Koskela et al., 2012). It is also worth noting that the perpetual flowering habit in most genotypes is linked to a loss of environmental sensitivity for flowering-promoting conditions. *F. vesca* accessions that flower seasonally require exposure to short photoperiods to induce flowering, whereas perpetual flowering accessions are day neutral (Koskela et al., 2012). Similarly, seasonal flowering *A. alpina* accessions require vernalization to flower, whereas perpetual flowering accessions can flower without being exposed to vernalization (Wang et al., 2009; Albani et al., 2012).

### Leaf Traits in the Annual *Cardamine hirsuta*

FLOWERING LOCUS C has been detected in QTL studies to underlie differences in leaf size and complexity in the annual Brassicaceae *Cardamine hirsuta* (Cartolano et al., 2015). Accessions with reduced expression of the *C. hirsuta FLC* ortholog (*ChFLC*) flower early and have leaves with more leaflets (Cartolano et al., 2015). Interestingly, *cis* polymorphisms in the nucleation region of *ChFLC* seem to be responsible for the differences in leaf complexity and flowering time (Cartolano et al., 2015). This effect of *ChFLC* on leaf shape cannot be uncoupled from flowering (Cartolano et al., 2015), suggesting that the leaf phenotype in *C. hirsuta* may be associated with flowering time.

### Pleiotropic Roles of FLC in Seed Traits in *A. thaliana* and *A. alpina*

Plant fitness can be influenced by seed traits such as seed dormancy and longevity. Seed dormancy is defined as the incapacity of seeds to germinate under favorable conditions and enables seeds to prevent germination outside the favorable growth season. Seed longevity determines how long seeds remain viable in the seed bank. Monocarpic plants are usually characterized by seeds with higher seed longevity, as seeds constitute their only option to persist in a certain habitat (Thompson et al., 1998).

**FIGURE 4:**
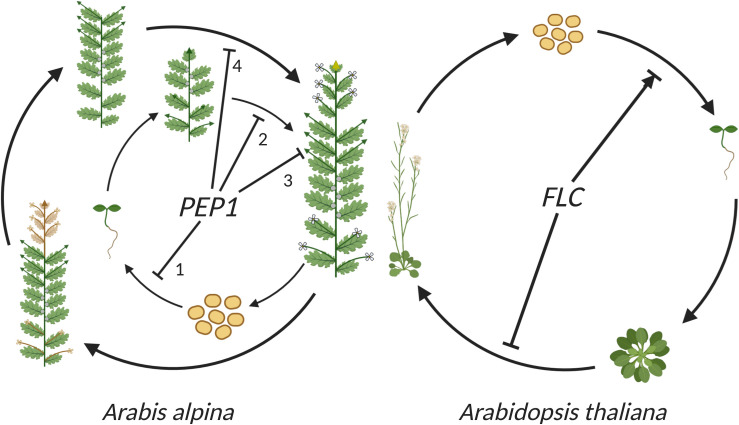
The role of *FLC* in the life cycle of perennial *A. alpina* and annual *A. thaliana*. In *A. alpina*, *PEP1* (*AaFLC*) regulates flowering in response to vernalization and contributes to the perennial life cycle by acting at different developmental stages and tissues. Starting from seeds, *PEP1* acts as a negative regulator of germination and an enhancer of seed longevity (1). Later on, *PEP1* represses flowering and ensures that plants will synchronize anthesis with favorable environmental conditions in the spring (2). *PEP1* also ensures that V3 axillary branches will remain vegetative after flowering (3). The role of *PEP1* in flowering and maintenance of vegetative development are repeated every year (4). In Arabidopsis, *FLC* acts as a floral repressor in accessions that require vernalization to flower. *FLC* also influences germination in *A. thaliana*, either positively or negatively depending on the environmental conditions and genetic background. Lines with (→) indicate positive influence and lines with (⊥) indicate negative influence. As in Figures 1, 3, *green arrows* represent vegetative branches, *yellow arrows* represent flowering branches and *gray circles* represent dormant V2 buds. Image created using BioRender.com.

In *A. thaliana*, several studies using the (low dormant) accessions Landsberg *erecta* (L*er*) and Columbia (Col) provided evidence that *FLC* enhances germination (Figure 4; Chiang et al., 2009; Auge et al., 2017; Blair et al., 2017). However, this positive effect of *FLC* on germination has not always been observed. Other studies (also using the L*er* and Col accessions) indicated that *FLC* plays a negative role in germination by enhancing seed dormancy (Figure 4; Chen et al., 2014; Chen and Penfield, 2018) or that it does not influence dormancy (Liu et al., 2011). These different results suggest a role of *FLC* in germination and dormancy in *A. thaliana* that could depend on environmental conditions and genetic backgrounds. This is also suggested by the observation that the germination phenotype of FLC depends on the dormancy level of seeds (Blair et al., 2017). In addition, these different observations could be explained by the function of other genes, such as *FT*, that control *FLC* expression during germination and independently affect seed germination (Auge et al., 2019). To further enhance the complexity by which these flowering time genes regulate seed traits, *FT* has also been shown to either enhance or reduce dormancy in different studies (Chiang et al., 2009; Chen et al., 2014; Chen and Penfield, 2018). Although complex, the observed phenotypes indicate a role for *FLC* and other flowering time genes in the control of germination. The exact role and mechanism of *FLC* in germination control is still unclear, but it seems likely to be influenced by environmental factors (in particular temperature) that vary between experiments. This is consistent with the observation that FLC is maternally controlled in its influence on dormancy (Chiang et al., 2009; Chen and Penfield, 2018). FLC is therefore likely to have a role in the translation of environmental conditions experienced by the mother plant into the dormancy level of its progeny seeds.

Although both flowering and germination should be properly timed to ensure reproductive success, the suppressive role of FLC on flowering seems to be more consistent among experiments and species, compared to its role in seed traits. An interesting difference between these two developmental transitions is that seed germination can be arrested and postponed for a year if conditions are unfavorable, which is not possible for flowering in monocarpic plants. Seed dormancy induction on the mother plant is also more sensitive to subtle differences in ambient temperature compared to flowering induction. Small differences in temperature before and during seed development can have a strong impact on seed dormancy (Springthorpe and Penfield, 2015). If FLC has a role in translating these subtle temperature differences to dormancy differences, it could explain the varying phenotypes between experiments. Another difference is that exposure to long-term low temperatures (below 10°C) downregulates *FLC* transcript accumulation and leads to flowering, whereas exposure of plants to mildly low temperatures (16 versus 22°C) before seeds are fully ripe can enhance *FLC* expression (Chen and Penfield, 2018). This suggests a different mechanism by which temperature regulates *FLC* expression, which could be related with its more flexible role in germination compared to flowering.

In annuals, FLC regulates both flowering time and germination traits and therefore constitutes a connecting factor between both traits. Different scenarios for this connection can be hypothesized which could be advantageous in different climates. The combination of late flowering and low dormancy could cause seeds to germinate in autumn and enable the plant to survive in a vegetative state during winter. Late flowering and high dormancy would delay germination till the next spring and enable plants to grow vegetatively during the year and flower the following spring. Early flowering and low dormancy would be advantageous in a climate with humid summers and enable two generations within one year. Early flowering and high dormancy would enable flowering and seed set in spring and delay germination until the next spring. In perennials, all these different scenarios are less relevant as plants can persist longer in their ecosystems.

In *A. alpina*, *PEP1* (*AaFLC*) also influences seed dormancy and seed longevity. The *pep1* mutant alleles have low dormancy and reduced longevity, suggesting that PEP1 positively regulates these traits (Figure 4; Hughes et al., 2019). The influence of *PEP1* on seed dormancy seems more consistent in *A. alpina* compared to that of *FLC* in *A. thaliana*. Although there has only been a single study of this trait in *A. alpina* until now, it showed a consistent role of PEP1 in greenhouse and garden experiments. The existence of many *A. alpina* accessions with non-functional *PEP1* alleles suggests an advantage of the combination of perpetual flowering and low dormancy in certain environments.

Overall, the role of FLC in the two major developmental transitions of plants and its adjustment to temperature give it an important regulatory role to determine the life cycle of Brassicaceae species. Additional functions of FLC as described in this review also suggest a link between the regulation of these developmental transitions and the architecture of the plant. This could have selective advantages in nature but also implies that breeding for altered flowering time in Brassicaceae species could have unexpected effects on seed traits and/or plant architecture or other still unknown trade-offs. Therefore, functional studies of this link can provide new insights in the relation between these traits and support breeding for crop adaptation to local and changing climates. Whereas *FLC* has been demonstrated to affect different traits in Brassicaceae, other genes are expected to fulfill its function in other plant families. For instance, in cereals the *VRN* genes have a similar role in the regulation of vernalization as *FLC* in the Brassicaceae. It would be of great interest to find out whether these genes also have similar roles in other traits that are regulated by FLC. Conservation of this link would indicate its general importance for survival and adaptation of plants to their environment.

## Author Contributions

All authors developed the concept and wrote the manuscript. NVdIT prepared the figures.

## Conflict of Interest

WJJS was employed by company Rijk Zwaan. The remaining authors declare that the writing of this review was conducted in the absence of any commercial or financial relationships that could be construed as a potential conflict of interest.
